# A Commentary on “Application of single-incision mini-sling surgery versus standard mid-urethral sling surgery in female stress urinary incontinence: a systematic review and meta-analysis of randomized controlled trials”

**DOI:** 10.1097/JS9.0000000000003409

**Published:** 2025-09-08

**Authors:** Rong Tang, Yuanzhi Li, Weimin Xie

**Affiliations:** a Department of Gynecology, Affiliated Nanhua Hospital, Hengyang Medical School, University of South China, Hengyang, Hunan Province, China; b Department of Neurosurgery, Affiliated Hengyang Hospital of Hunan Normal University & Hengyang Central Hospital, Hengyang, Hunan Province, China; c Department of Gynecology, Affiliated Hengyang Hospital of Hunan Normal University & Hengyang Central Hospital, Hengyang, Hunan Province, China

*Dear Editor,*

We read with considerable interest the systematic review and meta-analysis by Zhou *et al*^[1]^, recently published in the International Journal of Surgery. This work comprehensively compares the efficacy and safety of the vaginal single-incision mini-sling (SIMS) and standard mid-urethral sling (SMUS) in the management of female stress urinary incontinence (SUI) – a critical clinical question. The results demonstrated that, in the treatment of female SUI, SIMS is non-inferior to SMUS with respect to patient-reported cure rates, objective cure rates, quality of life, and postoperative complications. Furthermore, SIMS offers distinct advantages over SMUS in several key perioperative and postoperative outcomes, including shorter operative time, reduced length of hospital stay, less postoperative pain, lower incidence of organ injury, and decreased volume of postoperative bleeding. These findings support that SIMS represents a superior treatment modality for female SUI, as it achieves efficacy equivalent to SMUS while providing additional clinical benefits – rendering it of substantial clinical significance for the management of this condition. Nevertheless, certain details of the present findings warrant further investigation to validate and expand on these observations. This work has been reported in line with the TITAN criteria^[2]^.

First, the search strategy has inherent limitations. As detailed in Table 1, this systematic review and meta-analysis confined its literature searches to studies published between 2007 and 2024. Such a temporal restriction risks omitting relevant studies published prior to 2007, which may introduce bias and compromise the comprehensiveness and validity of the results. A core objective of literature searching is to identify all available published and unpublished studies that address the research question at hand. Generally, restricting searches by publication year is discouraged unless a robust theoretical or empirical rationale exists – for instance, when updating a previously conducted review^[3]^. Additionally, Table 1 indicates that searches in the Medline database were limited to English-language studies, which may result in a biased summary. Studies published in languages other than English, as well as those published in English, should be included in the conduct of all systematic reviews^[4]^.

Second, while the study used a random-effects model to account for between-study heterogeneity, it failed to fully explore the sources of substantial heterogeneity in some outcomes through subgroup analysis. For instance, operative time exhibited extremely high heterogeneity (I^2^ = 97%), and length of hospital stay also demonstrated high heterogeneity (I^2^ = 87%). Potential sources of such heterogeneity – including surgeon experience, variations in SIMS/SMUS device designs, and differences in BMI – were not systematically explored. This limits the interpretability of the pooled effect sizes and the generalizability of the results.

Third, the inclusion of TVT-Secur – a product withdrawn due to its high failure rates and associated complications – restricts the generalizability of the findings regarding the overall effectiveness of SIMS analyzed in this study^[5]^. This may also introduce bias into the conclusion that SIMS are less effective, necessitating a more cautious interpretation of such results. Given that multiple studies, including systematic reviews and meta-analyses published in reputable journals^[5,6]^, have underscored this concern, consideration should be given to either excluding TVT-Secur from the analysis or evaluating it separately. Notably, a 2014 systematic review and meta-analysis specifically conducted separate analyses of studies involving TVT-Secur for this very reason^[6]^. We therefore recommend that the authors either provide a meta-analysis table for SIMS excluding TVT-Secur as a reference for readers or categorize TVT-Secur separately from other SIMS products.

Finally, the study’s reliance on short-term follow-up compromises its capacity to assess long-term efficacy and safety. The mean follow-up duration is 13.42 months, with most outcomes analyzed based on 12-month data – yet the discussion rightly acknowledges that 3–5 years of follow-up is optimal for evaluating therapeutic durability and late-onset complications. Notably, as outlined in Table 1, five RCTs included in this meta-analysis have follow-up data spanning more than 3 years, and their long-term results should be presented in the text. Furthermore, if there is available useful data, meta-analysis should be performed for short-term and long-term results regarding patient-reported cure rates and objective cure rates, respectively. Ultimately, future RCTs incorporating extended follow-up periods would significantly strengthen the evidence base supporting the use of SIMS.

In conclusion, this meta-analysis provides valuable evidence confirming that SIMS is an effective and safe alternative to SMUS for female SUI, with superior perioperative performance. Nevertheless, this work has several limitations that warrant attention and improvement: a restricted literature search (constrained by both time frame and language), inadequate exploration of heterogeneity among included studies, the inappropriate inclusion of the TVT-Secur procedure, and a relatively short follow-up duration for outcome assessment.

## Data Availability

All data analyzed or generated during this study are included in the article.
